# Hippocampal tissue mechanics are sensitive to fluctuations in oestrogen across the rat oestrous cycle

**DOI:** 10.1093/braincomms/fcag228

**Published:** 2026-06-19

**Authors:** Katrina A Milbocker, L Tyler Williams, Sabrina S Vander Wiele, Emma D Zarate, Hillary Schwarb, Elijah E W Van Houten, Elise A Corbin, Anna Y Klintsova, Curtis L Johnson

**Affiliations:** Department of Biomedical Engineering, University of Delaware, Newark, DE 19716, USA; Department of Biomedical Engineering, University of Delaware, Newark, DE 19716, USA; Department of Radiology, Children’s Hospital of Philadelphia, Philadelphia, PA 19104, USA; Department of Biomedical Engineering, University of Delaware, Newark, DE 19716, USA; Department of Biomedical Engineering, University of Delaware, Newark, DE 19716, USA; Department of Psychological & Brain Sciences, University of Delaware, Newark, DE 19716, USA; Department of Psychology, University of Nebraska-Lincoln, Lincoln, NE 68588, USA; Université de Strasbourg, CNRS, INSERM, ICube, UMR7357, Strasbourg, France; Department of Biomedical Engineering, University of Delaware, Newark, DE 19716, USA; Department of Psychological & Brain Sciences, University of Delaware, Newark, DE 19716, USA; Department of Biomedical Engineering, University of Delaware, Newark, DE 19716, USA; Department of Psychological & Brain Sciences, University of Delaware, Newark, DE 19716, USA

**Keywords:** neuroimaging, magnetic resonance elastography, women’s brain health, biomarker development; oestrogen

## Abstract

Sex hormones exert profound effects on brain structure and function throughout the female reproductive lifespan. While previous studies have documented hormone-dependent changes in neuronal morphology and synaptic connectivity, the impact of oestrogen on brain tissue mechanical properties has been unexplored. Alterations to hippocampal microstructure are particularly sensitive to fluctuations in sex steroids, and studies in peripheral tissues reveal that oestrogen is a powerful modulator of tissue elasticity and organization. Here, we employed magnetic resonance elastography (MRE) to quantify whole-brain and hippocampal viscoelastic properties across the oestrous cycle in female rats *in vivo* and following targeted α oestrogen receptor antagonism. We demonstrate that hippocampal stiffness and damping ratio (viscosity) exhibit dynamic, phase-dependent fluctuations that correlate with hormonal status. Notably, hippocampal damping ratio, a measure of relative viscosity, increased by nearly 40% during the transition from proestrus to oestrus phases, mirroring the steep drop in oestrogen production that is characteristic of this phase progression. Pharmacological inhibition of oestrogen receptors with Fulvestrant prevented this cyclical change and instead maintained an elevated hippocampal damping ratio that was comparable to that measured during oestrus in normally cycling rats. Histological analyses revealed that these mechanical changes were accompanied by alterations in astrocyte and neuronal number, receptor expression and dendritic spine morphology in the CA1 subregion. Notably, oestrogen signalling was crucial for maintaining both astrocyte and neuronal populations and receptor density was elevated with the greatest effect on astrocytes (+48%) following Fulvestrant administration. These findings establish a mechanistic link between oestrogen fluctuations, cellular architecture and tissue mechanical properties in the female brain, offering novel insights into how reproductive endocrinology may influence hippocampal microstructure through mechanobiological pathways. Understanding these hormone-dependent mechanical changes may provide new therapeutic targets for addressing altered cognitive capacity associated with reproductive transitions and ageing in women.

## Teaser

First study linking non-invasive measurement of brain tissue mechanics to hormonal dynamics in female rats. Hippocampal damping ratio, relative viscosity, peaks during phases of low oestrogen via astrocyte and neuronal remodelling. Milbocker *et al*. demonstrate that magnetic resonance elastography scanning produces biomarkers for studying women’s brain health, validated with histology.

## Graphical Abstract

**Graphical Abstract:**
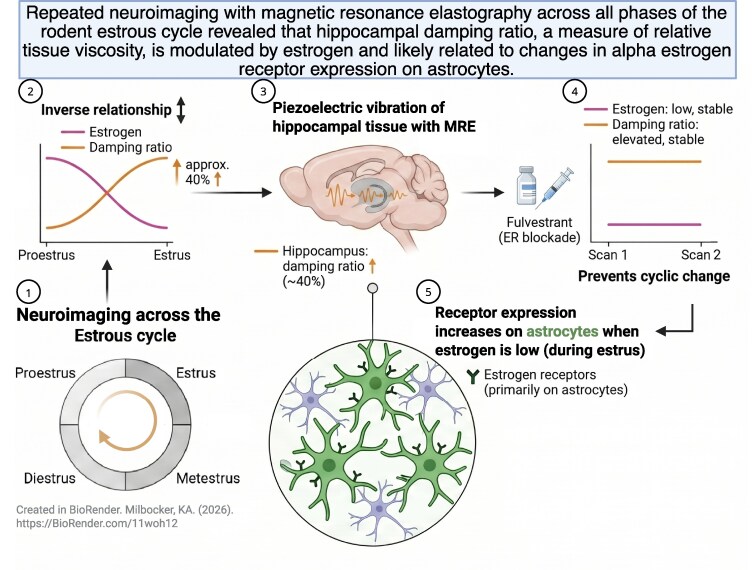

## Introduction

Fluctuations in oestrogen and progesterone play a vital role in women’s health from menarche to menopause, and the brain is an active endocrine organ in this complex hormonal interplay. These powerful sex hormones orchestrate a cascade of neurobiological changes spanning multiple scales—from microscopic remodelling of the epigenome, cellular architecture and nanoscale refinement of synaptic connectivity at dendritic spines^1-7^ to macroscopic alterations in brain volume and network connectivity patterns.^8-14^ Hormone-dependent neuroplastic adaptations occur in brain regions rich in oestrogen and progesterone receptors, including the hippocampus, amygdala and prefrontal cortex, where they modulate cognitive function, emotional processing and stress responses throughout the reproductive lifespan.^15-17^ Importantly, these hormonal fluctuations constitute essential signalling mechanisms that maintain neural homeostasis and confer cognitive flexibility.^18-22^ Understanding these complex neuroendocrine interactions provides crucial insights into neurological and psychiatric conditions which have a higher prevalence in women or a unique aetiology in the female brain.

The cellular basis for the effects of oestrogen and progesterone on the brain has been extensively studied in the rodent hippocampus, where hormone cycling drives remarkable structural plasticity and impacts memory.^14^ Several ground-breaking studies performed by McEwen and Woolley in the 1990s found that when oestrogen circulation was highest during the proestrus phase of the rodent oestrous cycle, the connectivity and signalling of hippocampal circuits were elevated, as evidenced by increased dendritic spine density, synaptogenesis and excitatory signalling.^1,2,22^ During the oestrus phase (after ovulation), when oestrogen is lowest, such features were retracted, and hippocampal astrocytic process extension increased, marking this more pro-inflammatory phase.^23^ While fluctuations in oestrogen orchestrate these microstructural changes, the rise of progesterone during late proestrus and slow decline during oestrus were found to coordinate the precise rhythmic timing of the changes such that they were complete by late oestrus.^2^

**Figure 1:**
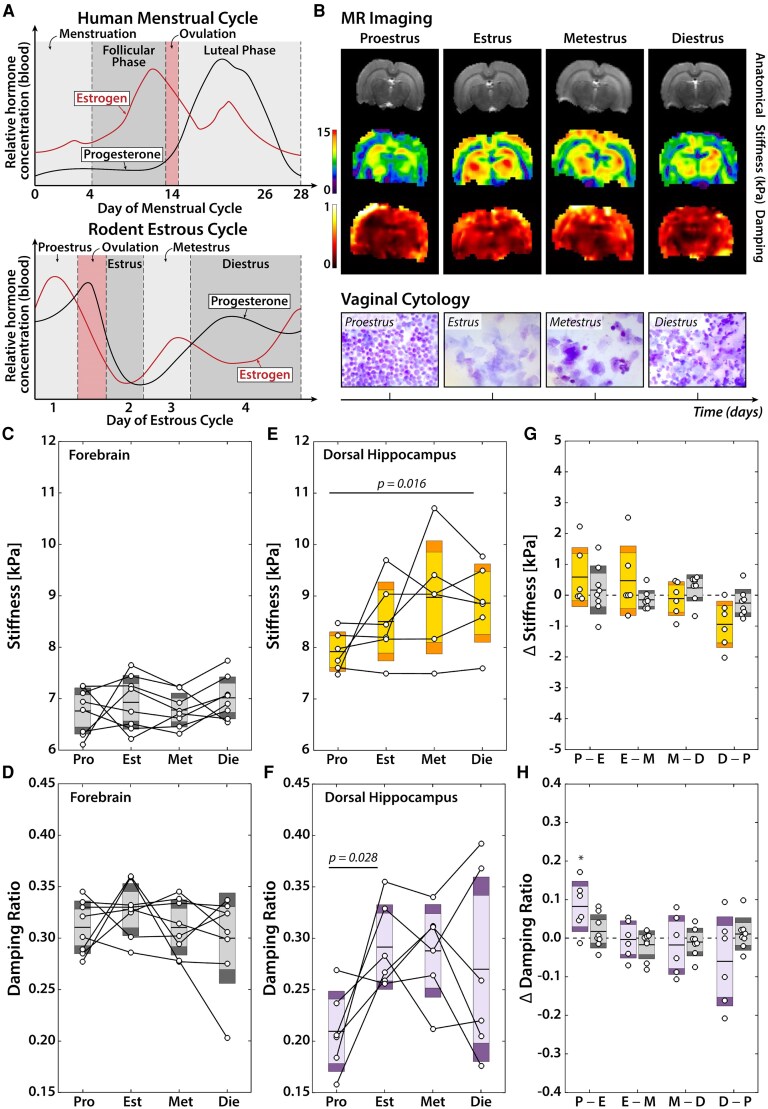
**Global and hippocampal tissue mechanics across the rat oestrous cycle.** (**A**) Fluctuations in oestrogen and progesterone production in humans (menstrual cycle) and rats (oestrous cycle), (**B**) Experimental timeline with representative images from vaginal cytology testing to confirm cycle phase. T2 anatomical and stiffness/damping ratio maps from *in vivo* MRE scanning at each cycle phase (stiffness scale bar cool 0—hot 15 kPa; damping ratio scale bar black 0—white 1), (**C**, **D**) Average *forebrain* stiffness values (kPa, F) and damping ratio for each rat compared using repeated measures ANOVA when measured across the four phases of the oestrous cycle (*n* = 8), Forebrain stiffness did not vary across cycle phases (*F*(3,21) = 0.871, *P* = 0.416, Greenhouse-Geisser corrected). Forebrain damping ratio also did not vary across cycle phases (*F*(3,21) = 1.473, *P* = 0.251). (**E**) Paired *t*-test revealed that the average *hippocampal* stiffness decreased between diestrus and proestrus phases (*t*(5) = 3.070, *P* = 0.016, *n* = 6), (**F**) Paired *t*-test revealed that the average *hippocampal* damping ratio increased between proestrus and oestrus phases (*t*(5) = −3.065, *P* = 0.028, *n* = 6), (**G**) Change in stiffness between consecutive phases in forebrain and dorsal hippocampus (*n* = 6). No other consecutive phase transitions were significant for forebrain stiffness (all *t*(7) ≤ 1.615, all *P* > 0.15, *n* = 8) or hippocampal stiffness (all *t*(5) ≤ 1.502, all *P* > 0.19), except the diestrus-to-proestrus transition reported in E. (**H**) Change in damping ratio between consecutive phases in forebrain and dorsal hippocampus (*n* = 6). No other consecutive phase transitions were significant for forebrain damping ratio (all *t*(7) ≤ 1.343, all *P* > 0.22, *n* = 8) or hippocampal damping ratio (all *t*(5) ≤ 1.333, all *P* > 0.24), except the proestrus-to-oestrus transition reported in F. Box plots depict mean (light shading) and standard deviation (darker shading) values while individual points represent average regional stiffness or damping ratio value per animal. Pro = proestrus, Est = oestrus, Met = metestrus, Die = diestrus, P-E = proestrus to oestrus transition, E-M = oestrus to metestrus transition, M-D = metestrus to diestrus transition, D-P = diestrus to proestrus transition.

Non-invasive neuroimaging has been a useful tool for identifying changes to regional brain structure with respect to fluctuations in sex steroids *in vivo* in both rodents and humans, where comparison of brain structure and function during the late-follicular and late-luteal phases provides a suitable analogue to the high versus low oestrogen phases in rodents. One distinct exception to the conservation of this hormonal cycle is that oestrogen peaks during ovulation in humans and rats do not undergo menses^14^ (Fig. 1A). Several studies have demonstrated that grey matter volume is highest during the late-follicular/poestrus^24,25^ phase and lowest during the early follicular/oestrus phase,^26,27^ with the greatest changes occurring in the hippocampus. In parallel to the cellular findings, it has been suggested that the temporal progression of oestrogen-mediated volumetric changes is also progesterone-dependent.^12,28^ However, not all regions follow this trend,^29,30^ indicating that region specificity is imperative for understanding the effects of oestrogen and progesterone on brain structure. In an exploratory longitudinal study where one female subject was repeatedly scanned using diffusion-weighted imaging across two menstrual cycles, it was found that hippocampal fractional anisotropy, an analogue measurement of tissue organization related to the restriction of water molecule movement within tissue, was highest during ovulation, suggesting that the hippocampus may be particularly organized when oestrogen is highest.^31^ These volumetric and microstructural alterations have functional consequences, as evidenced by shifts in cognitive strategies employed during different cycle phases. During high oestrogen phases, subjects (human and rodent) predominantly use hippocampal-based strategies for spatial navigation, while during low-oestrogen phases, striatal-based strategies dominate.^9,10,14^ With the rapid advancement of techniques to assess nuanced changes in brain structure and function over time with respect to circulating hormones, neuroimaging is becoming a promising tool for assessing women’s brain health.

Oestrogen-driven morphological changes in the brain are now understood to involve complex mechanobiological mechanisms activated by rapid non-genomic signalling cascades that alter cytoskeletal dynamics through Rho GTPases and actin-binding proteins, thereby modifying the mechanical properties of dendritic spines.^32,33^ Furthermore, oestrogen receptors interact with mechanosensitive adhesion molecules like integrins and cadherins, creating a bidirectional signalling system where hormonal and mechanical cues converge to regulate synaptic plasticity.^34,35^ In addition to neuronal microstructure, glia play a pivotal role in brain tissue rigidity. The rat hippocampus contains the highest density of astrocytes (>260 cells/mm^2^)^36^ compared to other grey matter structures, and these cells are imperative for neurovascular homeostasis,^37^ tissue rigidity,^38^ and glymphatic flow.^39^ The cytoplasmic membrane of an astrocyte is densely populated with α oestrogen receptors (αERs),^18,20^ making these cells adept at altering physiological and reactive status in response to variations in oestrogen, ultimately fine-tuning neuron excitability^18,20,40,41^ and glial phenotype, which may drive mechanobiological changes. In the absence of oestrogen, post-menopause, there is a rise in αERs,^15^ and this is suggested to play a prominent role in the nearly 2/3 increase in prevalence in Alzheimer’s Disease in women compared to men.^42,43^ These findings suggest that hippocampal tissue mechanics may provide an important window into oestrogen-associated changes to the brain. However, traditional neuroimaging techniques, volumetric MRI and diffusion-weighted imaging, do not test the biomechanics of brain tissue, ultimately creating a gap in our development of non-invasive imaging biomarkers for women’s brain health.

Magnetic resonance elastography (MRE) is an emerging neuroimaging technique that offers a unique opportunity to quantify tissue mechanical properties *in vivo* through the calculation of properties from the imaging of gently applied shear waves. Researchers have adopted MRE to study brain changes related to neurodevelopment, ageing and neurological conditions, providing insights that complement conventional structural and functional neuroimaging, such as volumetric analysis.^44^ Studies have further demonstrated that brain viscoelastic mechanical properties correlate with cognitive performance across the lifespan.^45-48^ Age-related decline in myelination, grey matter integrity and altered brain fluid dynamics have been proposed as mechanobiological links to memory decline in ageing. In addition to group differences, MRE is well-suited to determine subtle, individual variations in brain structure over time, positioning the technique as a valuable addition to neuroimaging protocols that aim to tackle more gradual changes to the brain within-subject. The damping ratio—a sensitive measure of the brain's viscous properties—provides a powerful metric for disentangling individual differences in neural mechanics that underlie specific cognitive functions, particularly within executive control networks and memory-dependent processes.^49^ Studies using shear wave elastography on peripheral tissues have demonstrated sensitivity to hormone fluctuations: alterations in breast tissue stiffness across the menstrual cycle,^50^ cervical and uterine tissues soften prior to parturition^51^ and dermal and uterine tissues soften post-menopause.^52^ While brain mechanics have been observed to vary based on sex, these findings are only at the group level.^53-55^

Here, we leverage MRE to investigate how brain tissue mechanics vary across the oestrous cycle in female rats, with particular focus on the hippocampus, to form a foundational basis for the use of MRE to study neurological conditions with a higher prevalence in women. Although limited in number, rodent MRE investigations have been instrumental in elucidating the molecular and cellular mechanisms that govern brain mechanical homeostasis.^56-62^ We hypothesized that rodent hippocampal mechanical properties would fluctuate between proestrus and oestrus phases, reflecting known cellular-level structural changes to astrocytes and neurons in response to the steep drop in oestrogen surrounding ovulation. Using a targeted receptor antagonist, we further examined the independent contribution of oestrogen signalling to these mechanical dynamics. These findings will provide new insight into the tissue-level manifestations of hormonal cycling on female brain mechanics and demonstrate the validity of MRE as a biomarker for women’s brain health, providing a new mechanobiology framework.

## Materials and methods

### Subjects and receptor antagonism

All experimental procedures were conducted in accordance with the National Institutes of Health Guide for the Care and Use of Laboratory Animals and ARRIVE guidelines and were approved by the University of Delaware Institutional Animal Care and Use Committee (Protocol #1409). Adult female Long-Evans rats (250–300 g, 3–4 months old) were housed in pairs under controlled temperature (22 ± 1°C) and humidity (50 ± 10%) conditions with *ad libitum* access to food and water. Animals were maintained on a reverse 12-h light/dark cycle (lights off at 0700 h). All efforts were made to minimize animal suffering and reduce the number of animals used. Oestrous phase identification via vaginal cytology with crystal violet staining was performed over two consecutive weeks prior to scanning between 13:00 and 15:00.^63,64^

Following oestrous cycle confirmation, female rats (*n* = 8) underwent three series of MRE scans. First, baseline scans were performed during each cycle phase (proestrus, oestrus, metestrus and diestrus, Fig. 1B). Second, rats received vehicle (saline, intraperitoneally) injections during diestrus and proestrus, followed by scans during proestrus and oestrus of the following cycle. Third, rats received Fulvestrant (αER antagonist, 0.2 mg in 1 mM DMSO, intraperitoneally, Millipore Sigma I4409)^65^ during diestrus and proestrus, acyclic behaviour was confirmed with vaginal swabbing for 7 consecutive days, and then 2 consecutive scans were performed. Fulvestrant is an αER antagonist with high permeability across the blood-brain barrier.^66-68^ All scans were conducted between 13:00 and 15:00. Between scanning series, rats were monitored via vaginal cytology for one complete oestrous cycle (4–5 days) to confirm normal cycling. Acyclicity was defined as either the persistence of one phase for ≥ 2 consecutive days or the absence of ≥ 2 phases for ≥ 2 consecutive days.

### Magnetic resonance elastography (MRE) scanning

All MRE scans were performed on a Bruker 9.4T BioSpec preclinical MR imaging system (Bruker Corporation, Billerica, MA). Rats were anaesthetized with 1–3% isoflurane administered via nose cone throughout each scanning session. Vitals were monitored using a respiration sensor and temperature probe, and body temperature was regulated by a liquid-heated animal cradle. The total scan time, including localization, MRE scan and anatomical scan, was approximately 45 min.

For the MRE scans, a non-magnetic, piezoelectric actuator (APA150M-NM, Cedrat Technologies, Meylan, France) delivered 700 Hz vibrations to the rodent’s brain via a custom 3D-printed bite bar.^61,62^ To ensure sufficient wave transmission between the rodent’s incisors and the brain, multiple bite bar sizes were utilized. Brain tissue displacement was on the order of 5–10 μm, consistent with previously reported rodent MRE setups.^61,69,70^ To ensure stability throughout the scan, the actuator was mounted to a custom 3D-printed bed extension. Positioning could be adjusted using guide rails, which slid along tubing slots in the animal cradle and were secured using mounting brackets on both sides. An MRE-EPI sequence developed in-house was used with 3D full-vector motion, positive and negative gradient polarities and four phase offsets for a total of 24 repetitions. MRE scans were acquired using the following imaging parameters: TE/TR = 60/4000 ms, FOV = 20 × 20 mm^2^, matrix = 80 × 80, slice thickness = 0.5 mm, slices = 40, averages = 12. A resolution of 0.25 × 0.25 × 0.5 mm^3^ was achieved in 19 min. Signal averages were used to ensure sufficient displacement data quality for stable mechanical property estimation with the non-linear inversion algorithm (NLI),^71^ as measured by the octahedral shear strain-based signal-to-noise ratio (OSS-SNR). Scans with OSS-SNR below the threshold of five were excluded. An example wave image is shown in Supplementary Fig. 1.

Mechanical properties were estimated from MRE displacement data using the isotropic, nearly incompressible, NLI algorithm formulated with no boundary conditions using a coupled adjoint field formulation to minimize error along the edges of the brain and between small brain regions.^72^ NLI has been described extensively in previous studies on both human and rodent MRE. In summary, it uses a coupled adjoint field formulation scheme to minimize the difference between measured and estimated displacement fields by iteratively updating maps of the complex shear modulus, $G^{*}=\;G^{\prime }+iG^{\prime \prime }$, via the storage modulus, $G^{\prime }$, and loss modulus, $G^{\prime \prime }$. The storage and loss moduli were then used to calculate shear stiffness $\mu \;=\;2|G^{*}{|}^{2}\;/\;(G^{\prime }+|G^{*}|)$, and damping ratio, $\xi \;=\;G^{\prime \prime }/2G^{\prime }$.^73,74^ Brains were segmented from the magnitude images using the SHERM automatic brain extraction tool,^75^ and voxel-wise mechanical property maps of the global forebrain were generated. The dorsal hippocampus was manually masked from the global forebrain. Slices at the extremities of the brain were excluded due to low wave motion, which resulted in poor mechanical property estimation. During image analysis, all experimenters were blind to treatment and oestrous phase.

### Immunohistochemistry

All rats were humanely euthanized and transcardially perfused with 100 ml of 0.1 M heparinised phosphate-buffered saline (PBS, pH = 7.20) solution, followed by 100 ml of 4% paraformaldehyde in PBS (pH = 7.20). The right hemisphere from each rat was post-fixed with a 30% sucrose in paraformaldehyde solution for immunocytochemical analysis, while the left hemisphere underwent Golgi–Cox impregnation for dendritic spine quantification. Additional cohorts were generated for histological analysis of brain tissue samples from all treatment groups during various phases of the oestrous cycle. A total of 40 additional rats were generated for histology and randomly selected to receive either saline treatment or left undisturbed (*n* = 10 saline/proestrus, 10 saline/oestrus, 10 undisturbed/proestrus and 10 undisturbed/oestrus). During tissue preparation and image analysis, all experimenters were blind to the rat treatment group or the oestrous phase.

Post-fixed 40 µm sections were prepared for immunocytochemical labelling of αER, astrocytes and neurons. Every eighth section through the rostro-caudal extent of the dorsal hippocampus was incubated in a blocking solution comprised of 0.5% Triton X-100 and 3% normal goat serum in 0.1 M Tris Buffer Saline (TBS) solution for 2 h. Free-floating tissue sections were then incubated overnight in a primary antibody solution containing polyclonal anti-GFAP^+^ (Progen GP52, 1:750), polyclonal anti-NeuN^+^ (Invitrogen, PA5-143567, 1:1500) and polyclonal anti-αER^+^ (Invitrogen, PA5-34577, 1:250) in blocking solution. The tissue was then triple-rinsed with 0.1 M TBS and incubated for 2 h in a secondary antibody solution containing AlexaFluor647 (Jackson Immuno, 106-605-003, 1:500), AlexaFluor594 (Jackson Immuno, 103-585-155, 1:500) and AlexaFluor488 (Jackson Immuno, 111-545-003, 1:500, Table 1). Sections were triple-rinsed in 0.1 M TBS, mounted on slides in deionized water, and then coverslipped with Gelvatol mounting medium. For Golgi–Cox impregnation of dendritic spines, 100 µm sections were collected, and the protocol for staining with the FD Rapid GolgiStain Kit (FD Neurotechnologies, PK401) was followed for perfused sections.

**Table 1:** Primary and secondary antibodies used for histological analysis of neurons and astrocytes

Target	Primary, manufacturer, dilution	Secondary, manufacturer, dilution
Astrocytes	guinea pig anti-GFAP^+^ polyclonal antibody (Progen GP52, 1:750)	Goat anti-guinea pig antibody conjugated with Alexa Fluor647 (Jackson Immuno, 106-605-003 at 1:500)
Neurons	Chicken anti-NeuN polyclonal antibody (Invitrogen, PA5-143567 at 1:1500)	Goat anti-chicken antibody conjugated with Alexa Fluor594 (Jackson Immuno, 103-585-155 at 1:500)
Alpha oestrogen receptors	Rabbit anti-ERa polyclonal antibody (Invitrogen, PA5-34577 at 1:250)	Goat anti-rabbit antibody conjugated with Alexa Fluor488 (Jackson Immuno, 111-545-003 at 1:500)

Unbiased stereology in Stereoinvestigator software (MBF Bioscience) with an optical fractionator probe^76^ was used to quantify neurons and astrocytes in each section. Each section was imaged using a Zeiss AxioImager M2 microscope with Colibri 7 LED illumination (Carl Zeiss AG, Oberkochen, Germany), resulting in six to eight sections quantified/rat. The optical fractionator parameters were set (section sampling fraction = 1/8, area sampling fraction = (150 μm × 150 μm counting frame)/(474 μm × 474 μm grid), dissector height = 20 μm, guard zone = 2 μm) after confirmation on a small subset of the samples that the coefficient of error never exceeded 0.1 for each cell type. Quantification of dendritic spines was performed using Neurolucida (MBF Bioscience)^2,77,78^ on a 15–20 μm segment of a dendrite from an apical pyramidal neuron in the stratum radiatum layer of hippocampal CA1. Images were acquired with a Zeiss Axioskop 2 Plus microscope with a 100× oil immersion objective.

### Statistical analysis

#### MRE scanning

We compared MRE outcomes within rats across all phases of the oestrous cycle to establish global (*n* = 8) and hippocampal (*n* = 6) changes to brain stiffness and damping ratio in the undisturbed condition. We used a repeated measure ANOVA with Greenhouse–Geisser and Bonferroni corrections for multiple comparisons in SPSS statistical software (IBM Corp). Regional and mechanical property measures were tested separately as dependent variables with time as the independent variable. Following oestrogen receptor antagonism, hippocampal mechanical properties during proestrus and oestrus were compared within-rat between scans following Fulvestrant and saline treatment using paired *t*-tests. To avoid bias, *a priori* criteria for exclusion (signal-to-noise ratio < 3.0 or excessive motion artefacts) were determined in advance, and a power analysis was conducted. Given the intended group size (*n* = 8), we were able to detect significant brain mechanical properties over time of medium effect size (*f* = 0.40) with 80% power (calculated using G*Power) using repeated measure ANOVA. All neuroimaging data are available in Supplementary Tables 1, 2 and 4.

#### Immunohistochemistry

Paired and independent samples *t*-tests were performed on cell number with and without αER to compare the effect of normal and induced oestrogen decline on receptor expression and overall cell population. A similar comparison was used to assess changes in apical dendritic spine density. *Post hoc* analysis with Bonferroni correction for multiple comparisons was used to determine specific group differences. Outliers outside of 2 IQR were discarded. All histology data are available in Supplementary Tables 3 and 5.

## Results

### Hippocampal mechanics change across the oestrous cycle

We hypothesized that the hippocampal damping ratio would be most affected between proestrus and oestrus as a function of the known changes to tissue microstructural organization accompanying transient oestrogen reduction. Paired *t*-tests were used to investigate whether forebrain or hippocampal stiffness and damping ratio varied between sequential cycle phases within-rat. Damping ratio increased by 38% between proestrus and oestrus (0.211 ± 0.016 versus 0.291 ± 0.017; *P* = 0.028; Fig. 1F), suggesting that in low oestrogen conditions, hippocampal tissue is more viscous or that ovulation has a significant effect on hippocampal tissue mechanics. No other cycle progressions exhibited a significant change in hippocampal damping ratio (all *P* > 0.05). Dorsal hippocampal stiffness declined by 10% between diestrus and proestrus (8.76 ± 0.74 versus 7.88 ± 0.36 kPa; *P* = 0.016; Fig. 1E); no other cycle phase progressions exhibited a significant change in stiffness (proestrus to oestrus; oestrus to metestrus; or metestrus to diestrus; all *P* > 0.05). It is possible that fluctuations in oestrogen and progesterone between diestrus and proestrus contributed to a decrease in hippocampal stiffness prior to the larger shift in oestrogen occurring between periovulatory phases. Global forebrain stiffness (average: 6.84 ± 0.40; all *P* > 0.05) and damping ratio (average: 0.316 ± 0.025; all *P* > 0.05) were not significantly different across cycle phases (Fig. 1C and D). Together, these data demonstrate that hippocampal tissue mechanics change across the rat oestrous cycle and appear to be most dynamic during periovulatory phases when the greatest change in oestrogen production occurs. We did not observe any significant differences in dorsal hippocampal volume between proestrus and oestrus (Supplementary Table 6). This observation aligns with existing MRI research on female rodent and human brain volumetrics and diffusivity, which similarly found no substantial structural variations to the whole brain associated with oestrogen fluctuations, with subtle changes to the hippocampus predominating and mirroring fluctuations in hormonal status.

**Figure 2:**
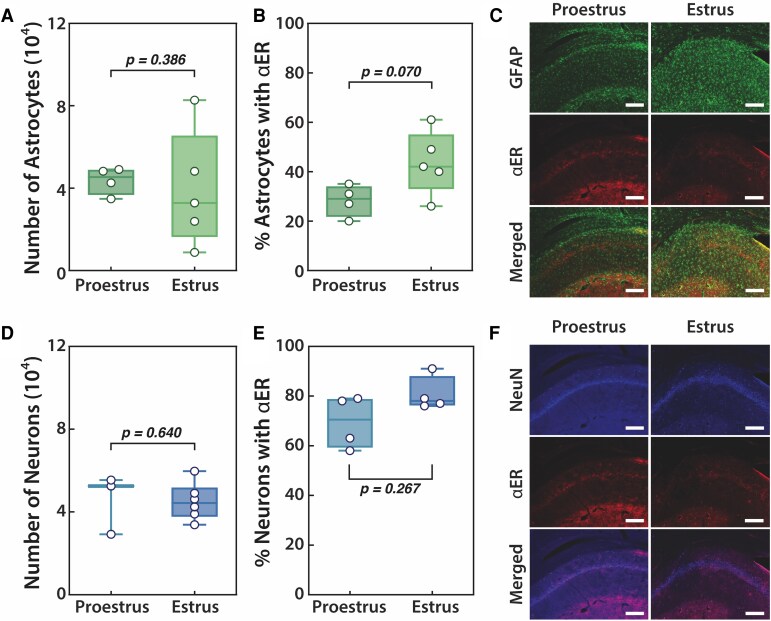
**Comparison of neuron and astrocyte number with α oestrogen receptor (αER) expression in the hippocampal CA1 subregion during proestrus and oestrus phases from undisturbed female rats.** Paired *t*-tests with Bonferroni correction for multiple comparisons revealed no significant differences in astrocyte number (**A**), neuron number (**D**), or in the percentage of astrocytes (**B**) and neurons expressing αER (**E**) between proestrus and oestrus phases (*P* > 0.05), (**C**) micrograph representations of astrocytes (GFAP^+^), αERs (αER^+^) and merged images, (**F**) micrograph representations of neurons (NeuN+), αERs (ER+) and merged images, scalebars = 200 μm. Sample size for each phase: proestrus *n* = 4, oestrus *n* = 5. Box plots error bars represent ± SEM. Individual data points in graphs A and D represent average cell number per animal, while data points in graphs B and E represent average number of co-labelled cells (GFAP^+^-αER^+^)/total GFAP^+^ cells per animal.

Although neuron and astrocyte density and reactivity are sensitive to gonadal hormone fluctuations and are known contributors to brain mechanobiology, it remains unclear how variations in these factors may be related to MRE outcomes. A separate cohort of female Long-Evans rats with confirmed oestrous cycling was perfused during proestrus or oestrus, and brain tissue was collected, post-fixed and stained with immunofluorescently tagged antibodies for mature neurons (NeuN), astrocytes (GFAP) and αERs. Cell number and αER expression were quantified using unbiased stereology with an optical fractionator probe through the rostral-caudal extent of hippocampal CA1, a robust and semi-automatic quantification method that estimated > 100 000 cells. The percentage of αER-expressing astrocytes trended upwards during oestrus compared to proestrus, though this difference was not statistically significant (28% ± 6% versus 44% ± 13%; *P* = 0.070; Fig. 2B and C). Activation of αERs on astrocytes tunes viscoelasticity of the cells,^38,41,79^ suggesting that transient changes in astrocytic αER density may play a key role in modulating the viscoelasticity of the surrounding environment. Additional differences in astrocyte number, neuron number and the percentage of αER-expressing neurons were not observed in CA1 between proestrus and oestrus (all *P* > 0.05; Fig. 2A, C, D–F).

### Effect of oestrogen receptor antagonism on hippocampal mechanical properties

**Figure 3:**
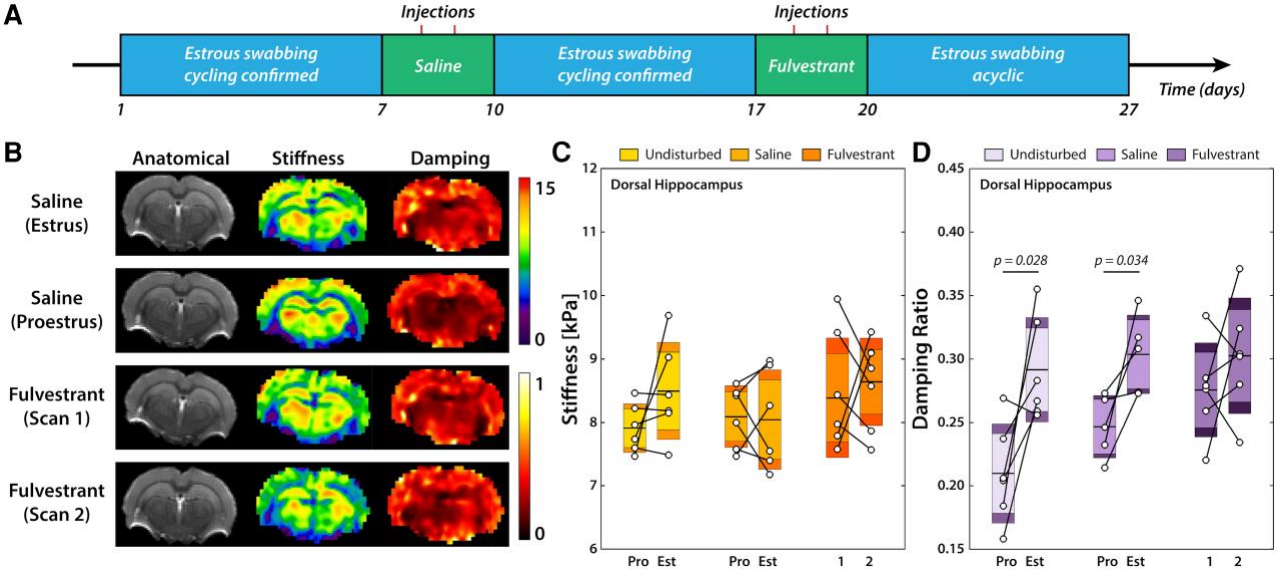
**Changes to hippocampal damping ratio are oestrogen-dependent.** (**A**) Experimental timeline depicting vaginal cytology to confirm normal cycling, vehicle saline treatment, repeated vaginal cytology to confirm normal cycling, Fulvestrant treatment and final vaginal cytology to confirm disrupted cycling (acyclic), (**B**) representative images from T2 anatomical scans and stiffness/damping ratio maps from data collected from *in vivo* MRE scanning (stiffness scale bar cool 0—hot 15 kPa; damping ratio scale bar black 0—white 1), (**C**) individual *t*-test with Bonferroni correction for multiple comparisons showed that average hippocampal stiffness is unchanged between proestrus and oestrus phases in undisturbed (*n* = 6, *P* > 0.05) and saline-treated rats (*n* = 6, *P* > 0.05) and between consecutive scans performed on Fulvestrant-treated rats (*n* = 6, *P* > 0.05), (**D**) individual *t*-test with Bonferroni correction for multiple comparisons confirmed that average hippocampal damping ratio increased between proestrus and oestrus phases in undisturbed (*P* = 0.028, *n* = 6) and saline-treated rats (*P* = 0.034, *n* = 6) but not across consecutive scans performed after Fulvestrant administration (*P*  *>* 0.05, *n* = 7). Box plots depict mean (light shading) and standard deviation (darker shading) values. Pro = proestrus, Est = oestrus, 1 = scan 1 after Fulvestrant treatment, 2 = scan 2 after Fulvestrant treatment. Individual data points represent average hippocampal stiffness or damping ratio per scan per animal.

To test the impact of αER signalling on brain tissue mechanics, rats from the previous imaging experiment received a systemic injection of vehicle saline, as a control for injection effects, and were scanned using MRE during periovulatory phases and then finally treated with Fulvestrant and scanned with MRE on 2 sequential days (Fig. 3A).

As expected, following the vehicle treatment control, hippocampal damping ratio continued to increase significantly between proestrus and oestrus phases, here by 24% (0.246 ± 0.025 versus 0.304 ± 0.031; *P* = 0.034; Fig. 3B and D), similar to the increase in damping observed between these phases in the undisturbed condition. Following αER antagonism with Fulvestrant, this cyclical change was prevented. Individual *t*-tests comparing the mean hippocampal damping ratio during either proestrus or oestrus after saline injection to the mean hippocampal damping ratio following Fulvestrant treatment, revealed that αER antagonism corresponded to an increase in hippocampal damping ratio such that it was comparable to that calculated from scans performed during oestrus (0.288 ± 0.028 versus 0.304 ± 0.031; *P* = 0.412), but not during proestrus (0.288 ± 0.028 versus 0.210 ± 0.031; *P* < 0.001, Fig. 3B and D). As expected, no significant difference in damping ratio was observed between consecutive days within the oestrus phase, even with Fulvestrant treatment (9% increase; 0.275 ± 0.037 versus 0.303 ± 0.046; *P* = 0.326, Fig. 3B and D). While oestrogen was the primary focus of this investigation, the effects of progesterone receptor antagonism were also considered, and those data are presented in the Supplementary Discussion and Supplementary Tables 7 and 8. We did not observe any significant differences in dorsal hippocampal volume between proestrus and oestrus following saline or Fulvestrant treatment, assessed from manual tracing of T2-anatomical scans (Supplementary Table 6).

Hippocampal stiffness did not significantly differ between proestrus and oestrus following vehicle saline (8.10 ± 0.49 versus 8.05 ± 0.78; *P* = 0.879) or Fulvestrant treatment (8.39 ± 0.94 versus 8.65 ± 0.69; *P* = 0.608, Fig. 3B and C), consistent with the observation in undisturbed rats. Independent *t*-tests comparing mean hippocampal stiffness during either proestrus or oestrus in vehicle-treated rats to mean hippocampal stiffness in Fulvestrant-treated rats revealed that αER antagonism had no effect on hippocampal stiffness compared to that measured during proestrus (8.54 ± 0.66 versus 8.11 ± 0.54; *P*  *=* 0.091) or oestrus (8.54 ± 0.66 versus 8.22 ± 0.74; *P*  *=* 0.113). These data demonstrate that oestrogen signalling is likely a key contributor to the viscosity but not the stiffness of the female rodent dorsal hippocampus during periovulatory phases, such that in typical and induced periods of low oestrogen, the dorsal hippocampal damping ratio increased.

**Figure 4:**
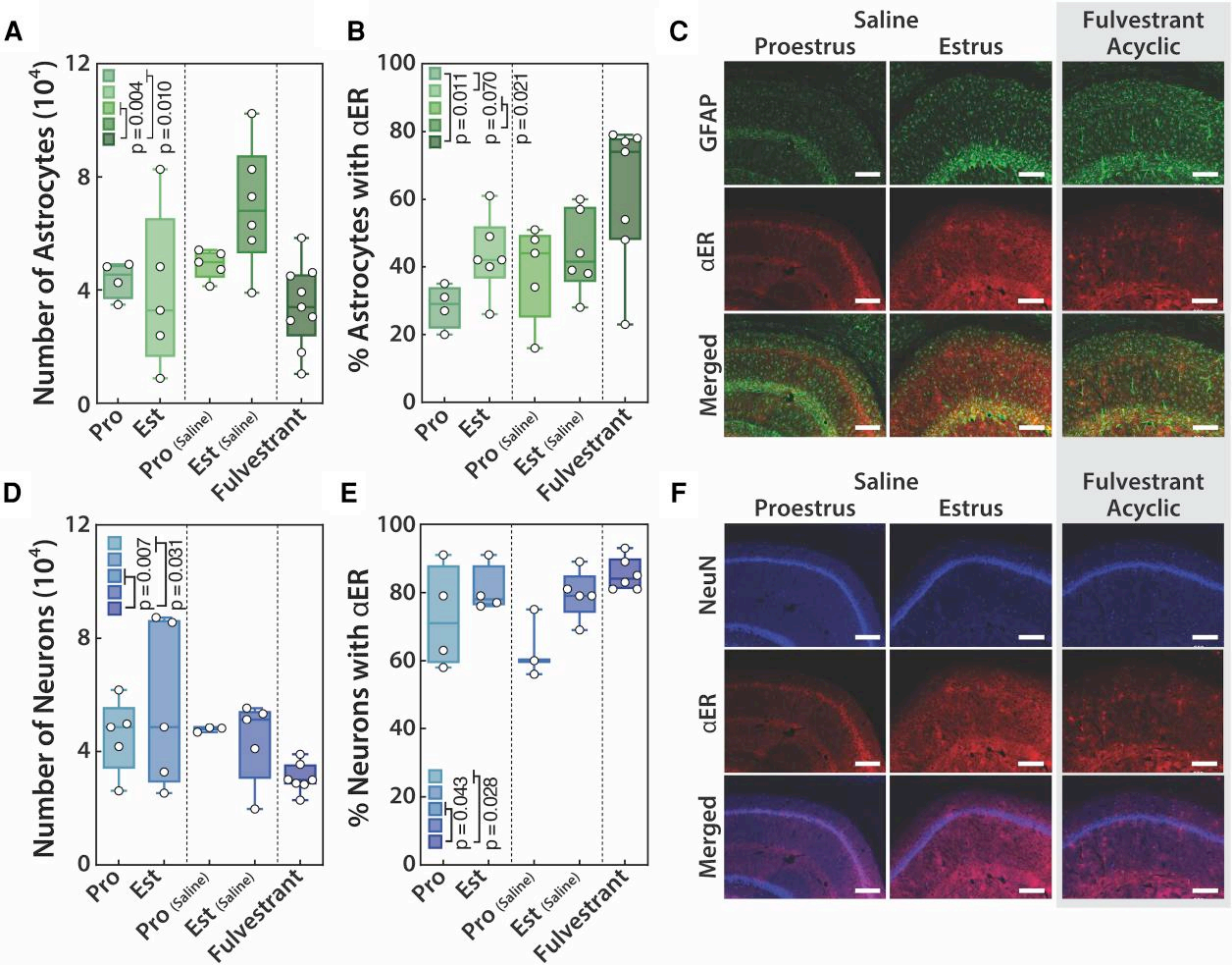
**Comparison of neuron and astrocyte number with α oestrogen receptor (αER) expression in the hippocampal CA1 subregion during proestrus and oestrus phases from undisturbed, saline-treated and Fulvestrant-treated female rats.** Paired *t*-tests with Bonferroni correction for multiple comparisons revealed that (**A**) the number of astrocytes is significantly reduced in Fulvestrant-treated rats compared to saline-treated (*P*  *=* 0.004) and undisturbed rats (*P*  *=* 0.010), and that (**B**) in the remaining astrocytes, αERs expression is increased following Fulvestrant administration. (**C**) Micrograph representations of astrocytes (GFAP^+^), αERs (ER^+^) and merged images, scalebars = 200 μm. Similarly, paired *t*-tests with Bonferroni correction for multiple comparisons revealed that (**D**) the number of neurons is significantly reduced in Fulvestrant-treated rats compared to saline-treated (*P*  *=* 0.007) and undisturbed rats (*P*  *=* 0.031), (**E**) in the remaining neurons, αERs expression is increased following Fulvestrant administration, (**F**) micrograph representations of neurons (NeuN^+^), αERs (ER^+^) and merged images, scalebars = 200μm. Sample size for each condition: Fulvestrant acyclic *n* = 9, saline proestrus *n* = 5, saline oestrus *n* = 6, undisturbed proestrus *n* = 4, undisturbed oestrus *n* = 5. Box plots error bars represent ± SEM. Individual data points in graphs A and D represent average cell number per animal, while data points in graphs B and E represent average number of co-labelled cells (GFAP^+^-αER^+^)/total GFAP^+^ cells per animal.

Comparison of astrocyte and neuronal number in hippocampal CA1 confirmed that αER antagonism had a significant effect on cellularity and receptor expression, potentially contributing to the observed changes in hippocampal damping ratio following Fulvestrant treatment. Tissue was collected from Fulvestrant-treated rats following MRE scanning. A second cohort of rats received vehicle treatment, analogous to that of the first cohort, and tissue was collected during proestrus or oestrus phases. Tissue collected from our undisturbed cohort of cycling rats (mentioned previously) in proestrus or oestrus phases was used to nullify the potential stress of intraperitoneal injection on histological results. Unbiased stereological estimation revealed that Fulvestrant treatment significantly reduced astrocyte and neuronal number compared to vehicle-treated rats (astrocytes: 34 515 ± 14 755 versus 60 258 ± 19 082, *P* = 0.004, Fig. 4A and C; neurons: 30 603 ± 5271 versus 45 509 ± 11 309, *P* = 0.007, Fig. 4D and F) and undisturbed rats (astrocytes: 34 515 ± 14 755 versus 49 042 ± 3.436, *P* = 0.010, Fig. 4A and C; neurons: 30 603 ± 5271 versus 50 980 ± 23 277, *P* = 0.031, Fig. 4D and F). There were no differences in mean astrocyte or neuronal number between proestrus and oestrus phases in vehicle-treated rats (astrocytes: 49 036 ± 5084 versus 69 606 ±21 835, *P* = 0.071; neurons: 38 279 ± 106 714 versus 45 924 ±23 113, *P* = 0.712) as previously observed in undisturbed rats.

Following disrupted αER signalling, the remaining astrocytes and neurons expressed greater αERs. Fulvestrant treatment increased αER expression on astrocytes and neurons compared to vehicle-treated rats (astrocytes: 62% ± 21% versus 42% ± 13%; *P* = 0.021; neurons: 85% ± 5% versus 77% ± 12%, *P* = 0.043) and undisturbed rats (astrocytes: 62% ± 21% versus 37% ± 13%; *P* = 0.011, Fig. 4B and C; neurons: 85% ± 5% versus 75% ± 11%, *P* = 0.028, Fig. 4E and F) irrespective of cycle phase. There were no cycle-specific changes to receptor expression observed on astrocytes or neurons between periovulatory phases in vehicle-treated rats (astrocytes: 73% ± 16% versus 80% ± 7%, *P* = 0.336, Fig. 4B and C; neurons: 73% ± 17% versus 83% ± 4%, *P* = 0.258, Fig. 4E and F) as previously observed in undisturbed rats. The effects of progesterone receptor antagonism on cell number and receptor expression were less clear (Supplementary Table 9). Moreover, given that Fulvestrant treatment reduced neuronal density in hippocampal CA1, pyramidal spine density was assessed with Golgi–Cox solution staining. It was anticipated that spine number would be reduced in Fulvestrant-treated rats when oestrogen decline was induced. Receptor antagonism reduced total spine density per 10 μm of apical dendrite in Fulvestrant-treated rats compared to undisturbed controls (10 ± 3 versus 13 ± 2, *P* = 0.051). Importantly, unlike in previously reported studies,^2,80^ a reduction in total spine density between proestrus and oestrus phases was not detected in undisturbed rats (13 ± 2 versus 12 ± 2, *P* = 0.360), likely due to uneven sample size per group (proestrus = 4, oestrus = 8). Disruption of αER signalling had a two-pronged effect: it was sufficient to reduce astrocyte and neuronal cellularity and caused a compensatory upregulation in astrocytic αER receptor expression in hippocampal CA1.

## Discussion

Our findings reveal that hippocampal tissue mechanics are dynamically regulated with αER signalling during the oestrous cycle when measured *in vivo* using MRE in the female rat. Hippocampal damping ratio exhibited high sensitivity to oestrogen fluctuations during periovulatory phases, with hippocampal damping ratio elevated by 38% in the oestrus phase when oestrogen is lowest. Fulvestrant-induced antagonism of αERs revealed that a steep and prolonged (+1 week) decline in oestrogen signalling is sufficient to elevate hippocampal damping ratio such that it was comparable to the mean damping ratio during oestrus; this mechanical signature parallels patterns seen in ageing and neurodegenerative conditions, where increased damping ratios correlate with cognitive decline.^46,81^

The concurrent alterations in damping ratio and cellular organization observed following Fulvestrant treatment reveal previously unrecognized connections between oestrogen receptor signalling and brain tissue mechanics. Damping ratio is a property that generally describes the attenuation of vibrational shear waves through tissue, with higher values representing greater viscosity and wave decay, and is typically considered to relate to the organization of cells or cellular features within a tissue.^44,49,56,82,83^ Changes in brain damping ratio have been associated with demyelination^60,61,84^ in preclinical rodent models of neuropathology, as myelin breakdown disrupts shear wave attenuation. However, the impact of gonadal hormone receptor expression in the context of brain tissue mechanics has been unexplored. Analysis of astrocytes and neurons within hippocampal CA1, the major endocrine-sensitive subregion of the dorsal hippocampus, revealed that induced disruption of oestrogen signalling was sufficient to reduce cell number and alter receptor expression on astrocytes. We found that administration of the αER antagonist Fulvestrant reduced the number of astrocytes and neurons in CA1 and led to compensatory elevations in αER expression on astrocytes. In undisturbed rats, αER expression on astrocytes trended upwards during the oestrus phase, convergent with the menopause literature, which has shown that αER expression is elevated during periods of low oestrogen.^15^ Total apical spine density on pyramidal neurons was reduced in Fulvestrant-treated rats compared to undisturbed rats, perhaps as a secondary compensatory mechanism related to tissue reorganization.

Glial cells, particularly astrocytes, significantly impact the brain’s viscoelastic properties through their extensive processes and contribution to the extracellular matrix. With reduced neuronal and astrocytic density, the network architecture becomes less densely interconnected, potentially increasing the damping ratio as the remaining tissue becomes less elastically efficient at propagating mechanical waves.^56,85,86^ αER expression on astrocytes is crucial for the neuroprotective effects of reactive astrogliosis,^18^ for the stiffening of astrocytes in response to environmental changes in overall tissue stiffness,^38,79^ and overall neuronal signalling in the hippocampus.^40^ These findings have relevance for ageing and neurodegenerative conditions, as aged, acyclic female rats show elevated astrocytic αER expression, contributing to neuronal estradiol desensitization post-menopause.^87^ Indeed, we observed that oestrogen signalling is crucial for neuronal maintenance or survival in this region in young adult rats. This pattern aligns with observations of increased αER expression in hippocampal CA1 tissue from postmenopausal AD patients^42^ and higher damping ratios in ageing adults’ hippocampi.^88^ Moreover, recent work demonstrates that changes in intracranial or intravascular pressure during ageing, in conjunction with rising GFAP levels in the blood, may be a viable biomarker for dementia in women^89^ as astrocytes mediate cerebral perfusion.^37^ These parallel changes in receptor expression, neuronal health and tissue mechanics suggest a potential mechanistic link between hormonal status, tissue properties and cognitive decline in females. We note that cell count alone does not capture all structural features that may contribute to tissue mechanics. Future studies should evaluate astrocyte tiling and branching in the hippocampus with respect to changes in damping ratio measured with MRE, as hippocampal astrocytes have shown enhanced branching with greater αER expression in low oestrogen conditions.^23,43,90,91^

The mechanosensitive nature of neurons and glia provides another angle from which to consider the role of dynamic hormone-dependent mechanical properties. Matrix stiffness significantly influences astrocyte reactivity, as demonstrated by *in vitro* studies using varying substrate stiffness.^38^ Astrocytes cultured on stiff hydrogels exhibit a larger proportion of active astrocytes with upregulated GFAP. Supporting this, white matter astrocytes are significantly softer than grey matter astrocytes, with the latter becoming softer following systemic immune activation, while white matter astrocytes appear to be more resistant to immune-induced changes to stiffness.^92^ The interaction between steroid hormones and the extracellular matrix adds another layer of complexity.

Our observations parallel findings in peripheral tissues, where mechanical properties fluctuate with hormonal status. Studies using shear-wave elastography have shown that breast glandular tissue and lower-limb muscles are stiffest during the follicular phase (low oestrogen) compared with the luteal phase (high oestrogen).^50,93^ Similarly, pregnancy-related hormonal changes drive cervical and uterine tissue softening through extracellular matrix reorganization.^51^ This is evidenced in decorin-null mice, where pregnancy temporarily resolves atypical collagen organization and promotes characteristic cervical tissue softening^35^ in preparation for parturition. Conversely, post-menopausal women exhibit decreases in tissue elasticity, which contribute to enuresis and wrinkle formation.^52^ Our results suggest similar principles may apply to brain tissue, with specific neurobiological mechanisms sensitive to sex steroids, and the relationship between hippocampal damping ratio and oestrogen levels may be extended to the post-menopausal female brain. We note that the data presented are from a limited number of rodents, but with dense, longitudinal measurements of the same animals, including mechanistic experimental manipulations. With a focus on repeated MRE scanning within-rat to increase statistical power, we aimed to show that MRE scanning can detect nuanced changes to brain tissue mechanics with respect to neuroendocrine fluctuations, thereby providing clearer insight into the capability of this tool to measure changes in female brain microstructure in rodents. The findings of the study can inform a follow-up study with a larger sample size to confirm the observed changes in hippocampal damping ratio with respect to oestrogen production.

Collectively, our findings reveal a complex interplay between sex steroid signalling, dynamic neurobiology and brain tissue mechanics that may have important implications for understanding both normal physiological function and disease processes. We demonstrate that oestrogen regulates hippocampal tissue properties through distinct cellular mechanisms. The parallel between our observations in the hippocampus and hormone-dependent mechanical changes in peripheral tissues suggests that tissue mechanical properties may be a fundamental target of steroid hormone action throughout the body. These hormone-dependent changes in tissue mechanics may represent a previously unrecognized mechanism by which sex steroids influence cognitive function through mechanobiological pathways. Understanding these mechanistic relationships should inform new therapeutic approaches for treating hormone-dependent cognitive decline and neurodegenerative conditions.

## Supplementary Material

fcag228_Supplementary_Data

## Data Availability

The experimental data for this manuscript are available in the Supplementary Information.
