# Supplementary material for: Hippocampal tissue mechanics are sensitive to fluctuations in oestrogen across the rat oestrous cycle
Source: Brain Commun. 2026 Jun 19;8(4):fcag228. doi: 10.1093/braincomms/fcag228 (PMC13450688; doi:10.1093/braincomms/fcag228)
Supplement: Supplementary file 1 [file fcag228_supplementary_data.pdf]

Hippocampal tissue mechanics are sensitive to fluctuations in estrogen across the rat estrous cycle

Supplementary Information

Supplementary Data

Supplementary Table 1. Average *forebrain* stiffness (μ) and damping ratio (ξ) values calculated from MRE scanning during each phase of the estrous cycle in undisturbed rats. Data availability for Figure 1.

Rat ID	*Proestrus*		*Estrus*		*Metestrus*		*Diestrus*	
	μ	ξ	μ	ξ	μ	ξ	μ	ξ
2-1	7.24	0.277	7.25	0.359	6.92	0.277	7.43	0.275
2-2	6.94	0.285	6.75	0.325	6.62	0.345	7.08	0.306
2-3	7.11	0.301	7.44	0.286	7.23	0.278	7.74	0.203
2-4	6.30	0.345	7.19	0.301	6.76	0.302	7.06	0.324
2-5	6.11	0.330	7.65	0.330	7.22	0.334	6.54	0.331
2-6	7.25	0.289	6.22	0.360	6.71	0.294	6.61	0.337
2-7	6.78	0.321	6.42	0.328	6.46	0.314	6.77	0.299
2-8	6.36	0.335	6.51	0.332	6.32	0.338	6.90	0.324

Supplementary Table 2. Average *hippocampal* stiffness and damping ratio values calculated from MRE scanning during each phase of the estrous cycle in undisturbed rats. Missing values are due to poor data quality with OSS-SNR < 4. Data availability for Figure 1.

Rat ID	*Proestrus*		*Estrus*		*Metestrus*		*Diestrus*	
	μ	ξ	μ	ξ	μ	ξ	μ	ξ
2-1	8.23	0.237	8.19	0.283	10.700	0.21	9.760	0.22
2-2	7.47	0.204	9.69	0.259	9.030	0.31	9.490	0.21
2-3								
2-4								
2-5	7.74	0.158	9.03	0.267	9.030	0.31	8.840	0.26
2-6	8.47	0.206	8.44	0.329	9.400	0.29	8.880	0.37
2-7	7.60	0.269	7.49	0.256	7.487	0.26	7.590	0.18
2-8	7.97	0.184	8.16	0.355	8.160	0.34	8.580	0.39

Supplementary Table 3. Neuron and astrocyte number and αER expression obtained from unbiased stereological estimation in dorsal hippocampal CA1. Outliers outside of 2 IQR discarded. Data availability for Figure 2.

Rat ID	*Treatment*	*Phase*	*# Astrocytes*	*% Astrocytes with αER*	*# Neurons*	*% Neurons with αER*
3-18	Undisturbed	Estrus	32785	61	46157	79
3-19	Undisturbed	Proestrus	34798	31	29190	63
3-20	Undisturbed	Estrus	82681	49	38968	77
3-23	Undisturbed	Proestrus	48192	35	52484	78
3-25	Undisturbed	Proestrus	42563	27		79
3-26	Undisturbed	Estrus	23870	26	42419	91
3-27	Undisturbed	Estrus	48192	40	59674	76

3-28	Undisturbed	Estrus			49033	
3-30	Undisturbed	Proestrus	49033	20	55360	58
3-34	Undisturbed	Estrus	8786	42	33791	

Supplementary Table 4. Average *hippocampal* damping ratio and stiffness values calculated from consecutive MRE scans after vehicle saline and Fulvestrant treatment. Missing values are due to poor data quality with OSS-SNR < 4. Data availability for Figure 3.

Rat ID	*Saline-treated*		*Saline-treated*		*Fulvestrant-treated*		*Fulvestrant-treated*	
	Proestrus	*Estrus*	*Proestrus*	*Estrus*	*Scan 1*	*Scan 2*	*Scan 1*	*Scan 2*
	ξ		μ		ξ		μ	
2-1	0.232	0.346			0.259	0.280	7.79	9.43
2-2	0.268	0.274	8.430	8.980			9.95	8.86
2-3	0.214	0.273	8.000	7.180				
2-4	0.273	0.308	7.580	7.400	0.285	0.304	7.98	7.57
2-5			8.620	8.909	0.276	0.234	9.42	8.58
2-6	0.246	0.317	7.470	8.270	0.334	0.302	8.44	7.87
2-7			8.470	7.550	0.220	0.324	7.58	9.10
2-8					0.279	0.371	7.58	9.10

Supplementary Table 5. Neuron and astrocyte number and αER expression obtained from unbiased stereological estimation in dorsal hippocampal CA1 after vehicle saline and Fulvestrant treatment. Missing values are due to poor fixation or outliers outside of 2 IQR. Data availability for Figure 4.

Rat ID	*Treatment*	*Phase*	*# Astrocytes*	*% Astrocytes with αER*	*# Neurons*	*% Neurons with αER*
2-1	Fulvestrant	n/a	46123	54		
2-2	Fulvestrant	n/a	30484	77	28759	
2-4	Fulvestrant	n/a	10353		28327	89
2-5	Fulvestrant	n/a	39255	79	30053	85
2-6	Fulvestrant	n/a	17932		22799	83
2-7	Fulvestrant	n/a	58442	74	38961	93
2-8	Fulvestrant	n/a	33929	78		
3-3	Saline	Estrus	62981	57		
3-4	Fulvestrant	n/a	44922	23	35416	81
3-5	Fulvestrant	n/a	29190	48	29909	81
3-7	Saline	Proestrus	52772	34		
3-8	Saline	Proestrus	47252	44	48370	60
3-9	Saline	Estrus	57597	44	19712	79
3-10	Saline	Proestrus	49824	16	46793	56
3-11	Saline	Estrus	102380	39	55360	89
3-13	Saline	Estrus	82681	38	53347	81
3-14	Saline	Proestrus	54066	48		
3-15	Saline	Estrus	38968	60	40981	79
3-16	Saline	Proestrus	41268	51	48171	75
3-17	Saline	Estrus	73047	28	51334	69
3-18	None	Estrus	32785	61	87282	79
3-19	None	Proestrus	34798	31	49771	63
3-20	None	Estrus	82681	49	85557	77

3-23	None	Proestrus	48192	35	61771	78
3-25	None	Proestrus	42563	27	26093	79
3-26	None	Estrus	23870	26	48602	91
3-27	None	Estrus	48192	40	25294	76
3-30	None	Proestrus	49033	20	41700	58
3-34	None	Estrus	8786	42	32750	

I. <u>Data availability for dorsal hippocampal volume across periovulatory phases</u>

Dorsal hippocampal volume was measured from T2 anatomical scans collected during MRE scan acquisition in undisturbed, saline-treated, and Fulvestrant-treated rats. Unlike in previous human and rodent studies, we did not observe any significant differences in dorsal hippocampal volume at any point. Paired t-tests were used to compare hippocampal volume within treatment paradigm.

Supplementary Table 6. Volume analysis from T2 anatomical scans from each rat following each treatment phase. Missing values are due to outliers outside of 2 IQR.

Rat ID	***Undisturbed***		***Saline-treated***		***Fulvestrant-treated***	
	Proestrus	***Estrus***	***Proestrus***	***Estrus***	***Scan 1***	***Scan 2***
	Volume (mm^3)		Volume (mm^3)		Volume (mm^3)	
2-1	13.281	14.781	16.469	15.781	15.594	11.875
2-2	15.031	15.719	15.219	11.563	19.750	15.656
2-3	9.625	12.406	13.125	10.781		
2-4	12.031	9.063	15.813	14.000	13.844	11.188
2-5	11.719	12.875	13.156	15.750	13.844	12.688
2-6	15.594	12.688	12.344	16.063	14.156	13.250
2-7	10.844	9.250	13.344	9.563	11.344	11.531
2-8	13.094	13.719	13.625		10.531	13.031

Supplementary Note and Data

MRE and cell data availability for post-RU486 treatment (progesterone receptor antagonism)

MRE Methods: To determine if progesterone receptor (PR) antagonism had a different effect on brain mechanics, a second cohort of rats (n = 8) was scanned in a similar manner to that described for the Fulvestrant antagonism experiment. Rats received two doses of RU486 (PR antagonist, 2mg in a stock solution, intraperitoneally, Fisher Scientific 14-791-00) at 12:30 and 17:30 during proestrus. RU486 was mixed in a stock solution containing 80% sesame oil, 15% benzyl benzoate and 5% ethanol and administered as a 0.2ml injection[1,2]. Vaginal swabbing was performed to confirm atypical cycling. MRE scans were performed on consecutive days after RU486 administration. Following scanning, rats were humanely euthanized and brain tissue was extracted for histology. Two histology-only cohorts of rats were generated to collected brain tissue during proestrus and estrus following vehicle injection (n = 10). Paired t-tests were used to analyze changes

between consecutive scans, while independent samples t-tests were employed to compare RU486-treated animals against saline controls.

Histology Methods: Immunofluorescent staining was performed on tissue samples as described in *Methods*; albeit, PR receptors were visualized with the primary polyclonal anti-PR^+ antibody and AlexaFluor488 (**Supplementary Table 7**). Unbiased stereological quantification of neurons, astrocytes and PR expression was performed in accordance with that described in *Methods*. Histological data were compared using unpaired t-tests with Bonferroni correction.

Supplementary Table 7. Primary and secondary antibodies required for histology.

Target	***Primary, Manufacturer, Dilution***	***Secondary, Manufacturer, Dilution***
Astrocytes	guinea pig anti-$GFAP^+$ polyclonal antibody (Progen GP52, 1:750)	Goat anti-guinea pig antibody conjugated with Alexa Fluor647 (Jackson Immuno, 106-605-003 at 1:500)
Neurons	Chicken anti-NeuN polyclonal antibody (Invitrogen, PA5-143567 at 1:1500)	Goat anti-chicken antibody conjugated with Alexa Fluor594 (Jackson Immuno, 103-585-155 at 1:500)
Progesterone receptors	Rabbit anti-PR polyclonal antibody (Sigma-Aldrich, SAB4502184 at 1:300)	Goat anti-rabbit antibody conjugated with Alexa Fluor488 (Jackson Immuno, 111-545-003 at 1:500)

Supplementary Figure 1. Example wave image from MRE scanning.

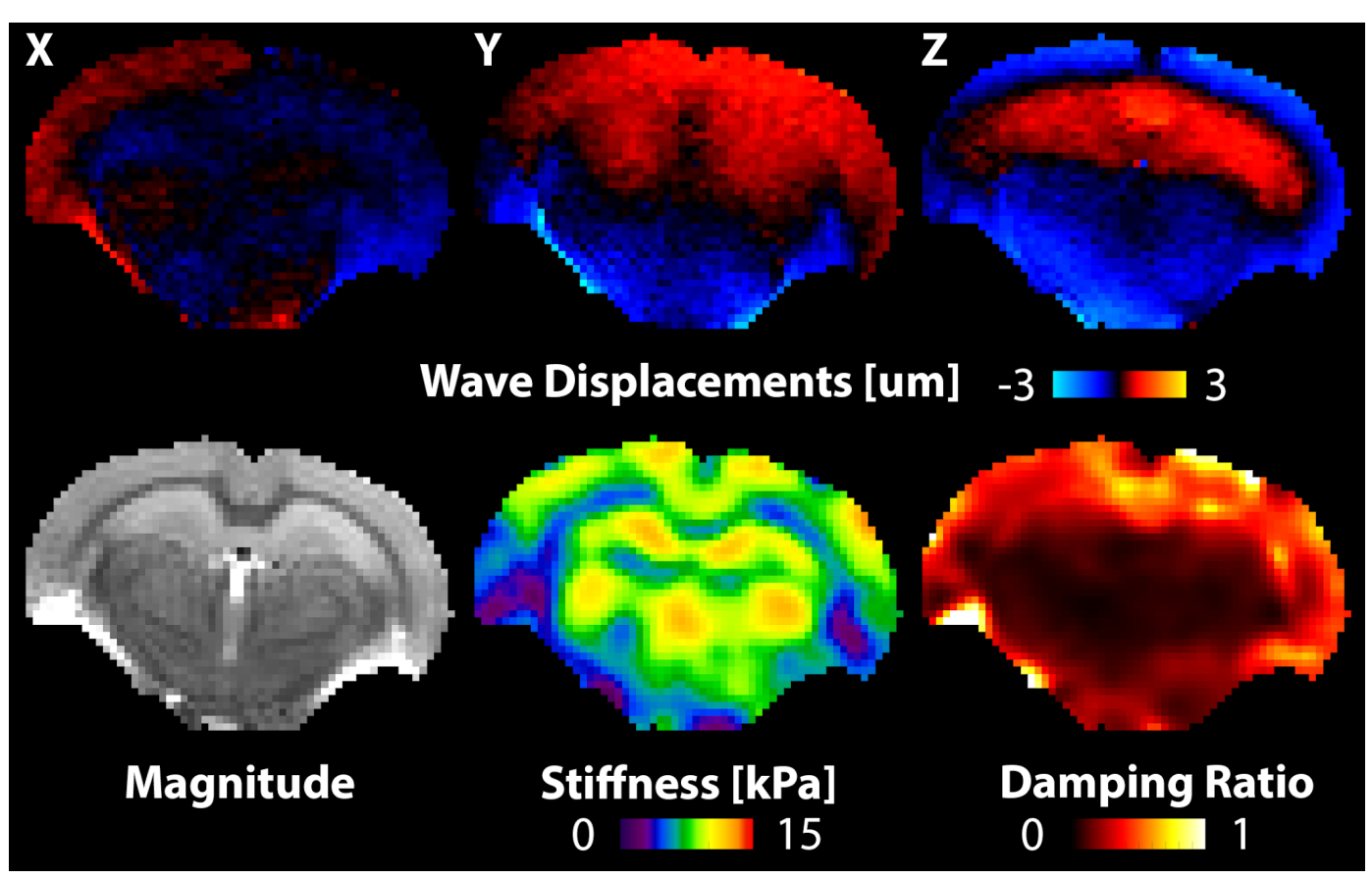

Supplementary Figure 1. Wave displacement in the X, Y, and Z directions. Example magnitude, stiffness, and damping ratio maps calculated from the wave propagations.

Supplementary Discussion

Progesterone levels during proestrus coordinate the drop in estrogen that occurs following ovulation in female rats, and while progesterone is not necessary to maintain cycling in female rats, temporal changes in synaptic composition during periovulatory phases are coordinated by the rise in progesterone[3]. Given the critical role of estrogen in fluctuations to hippocampal tissue mechanics, we expected that progesterone would also alter tissue viscosity across periovulatory phases.

Hippocampal damping ratio did not vary significantly between consecutive scans captured after RU486 antagonism (19% decrease; 0.293 ± 0.044 vs. 0.235 ± 0.044; $p = 0.118$). However, independent samples t-tests comparing average hippocampal damping ratio after PR antagonism to saline-treated rats (from the Fulvestrant experiment) revealed that damping ratio declined following progesterone-related disruption such that it was comparable to hippocampal damping ratio during proestrus (0.264 ± 0.010 vs. 0.210 vs. 0.040; $p = 0.001$) but not estrus in saline-treated rats (0.264 ± 0.010 vs. 0.292 vs. 0.041; $p = 0.112$). Hippocampal stiffness did not change between consecutive scans after RU486 treatment (8.01 ± 0.85 vs. 8.37 ± 0.73; $p = 0.586$), and average hippocampal stiffness after RU486 treatment was not significantly different from stiffness values during proestrus (8.19 ± 0.81 vs. 8.10 ± 0.49; $p = 0.693$) or estrus in saline-treated rats (8.19 ± 0.81 vs. 8.05 ± 0.78; $p = 0.692$). See **Supplementary Table 8** for hippocampal damping ratio and stiffness data.

Supplementary Table 8. Average *hippocampal* damping ratio and stiffness values calculated from consecutive MRE scans after RU486 treatment.

Rat ID	***RU486-treated***			
	Scan 1	***Scan 2***	***Scan 1***	***Scan 2***
	ξ		μ	
6-1	0.305	0.231	7.36	8.79
6-2				
6-3				
6-4	0.255	0.240	7.56	9.30
6-5	0.321	0.227	7.43	8.23
6-7	0.260	0.252	7.61	8.46
6-8	0.362	0.178	9.44	7.12
6-16	0.257	0.282	8.65	8.29

PR expression in the hippocampus does not change across the estrous cycle in rats[4]. We predicted that PR antagonism would increase PR receptor expression on astrocytes in hippocampal CA1. However, it was discovered that PR antagonism had no effect on PR expression on $GFAP^{+}$ astrocytes in CA1 compared to saline-treated rats (52% ± 13% vs. 56% ± 7%; $p = 0.397$) and undisturbed rats (52% ± 13% vs. 56% ± 9%; $p =$

0.581). There were no differences in the percentage of PR^+ astrocytes between cycle phases in saline-treated (60% ± 5% vs. 54% ± 8%; $p = 0.124$) and undisturbed rats (60% ± 4% vs. 53% ± 11%; $p = 0.236$). Convergent, the number of astrocytes in CA1 was reduced in RU486-treated rats compared to saline-treated rats irrespective of phase (38,893 ± 6,529 vs. 57,961 ± 24,239, $p = 0.027$). We note that control data were collected from undisturbed and saline-treated rats across two separate experimental cohorts: one cohort with Fulvestrant-treated rats and another with RU486-treated rats. The control groups across these independent cohorts exhibited consistent results, though each control group was analyzed only within its respective experimental cohort. The percentage of PR^+ neurons increased during estrus compared to proestrus in saline-treated rats (54% ± 1% vs. 46% ± 4%, $p = 0.028$), however this cycle-dependent increase was not observed in undisturbed rats (50% ± 15% vs. 38% ± 4%, $p = 0.228$). This suggests that saline treatment had a mild stress effect on PR expression. PR expression was significantly enhanced following RU486 treatment compared to saline-treated rats in both phases (83% ± 16% vs. saline pro = 46% ± 4%, $p = 0.003$ or saline est = 54% ± 1%, $p = 0.003$) and undisturbed rats (83% ± 16% vs. 46% ± 13%, $p < 0.001$). RU486-treated rats did not change in neuron number compared to saline-treated rats (42,233 ± 3,531 vs. 53,617 ± 21,584, $p = 0.326$), or undisturbed rats (42,233 ± 3,531 vs. 45,231 ± 10,090, $p = 0.582$). See **Supplementary Table 9** for all histological data.

Supplementary Table 9. Neuron and astrocyte number and PR expression obtained from unbiased stereological estimation in dorsal hippocampal CA1 after vehicle saline and RU486 treatment. Missing values are due to poor fixation or outliers outside of 2 IQR.

Rat ID	Treatment	Phase	# Astrocytes	% Astrocytes with PR	# Neurons	% Neurons with PR
3-1	Saline	Estrus		58	66145	54
3-3	Saline	Estrus	89583	56	59386	46
3-7	Saline	Proestrus	80668	62	95133	49
3-8	Saline	Proestrus	66145	52	68899	49
3-9	Saline	Estrus	44576	45	40837	54
3-10	Saline	Proestrus	76498	63	57805	44
3-11	Saline	Estrus	49752	45		
3-13	Saline	Estrus	39399	50	44144	53
3-14	Saline	Proestrus	29765	58		
3-15	Saline	Estrus	31347	66	31634	55
3-16	Saline	Proestrus	34942	65	54497	41
3-17	Saline	Estrus	51623	56	17687	52
3-18	None	Estrus	29190	65	46157	45
3-19	None	Proestrus	36811	59	29190	40
3-20	None	Estrus	52628	61	38968	39
3-21	None	Estrus	40406	63	42419	32
3-23	None	Proestrus	46301	64	52484	33
3-25	None	Proestrus	35804	57	55360	40
3-26	None	Estrus	36811	41	59674	48
3-27	None	Estrus	45582	47	49033	60

3-34	None	Estrus	37818	43	33791	73
6-1	RU486	n/a	40550	58	42994	63
6-2	RU486	n/a	33016	52	38873	85
6-4	RU486	n/a	47394	63	46861	100
6-7	RU486	n/a	34613	35	40205	86
6-8	RU486	n/a				

Supplementary References

1. Edwards HE, Epps T, Carlen PL, MacLusky NJ. Progestin receptors mediate progesterone suppression of epileptiform activity in tetanized hippocampal slices in vitro. *Neuroscience*. 2000;101(4):895-906. doi:10.1016/S0306-4522(00)00439-5
2. McEwen BS, Woolley CS. Estradiol and progesterone regulate neuronal structure and synaptic connectivity in adult as well as developing brain. *Exp Gerontol*. 1994;29(3-4):431-436. doi:10.1016/0531-5565(94)90022-1
3. Woolley CS, McEwen BS. Roles of estradiol and progesterone in regulation of hippocampal dendritic spine density during the estrous cycle in the rat. *Journal of Comparative Neurology*. 1993;336(2):293-306. doi:10.1002/CNE.903360210
4. Guerra-Araiza C, Villamar-Cruz O, González-Arenas A, Chavira R, Camacho-Arroyo I. Changes in Progesterone Receptor Isoforms Content in the Rat Brain During the Oestrous Cycle and After Oestradiol and Progesterone Treatments. *J Neuroendocrinol*. 2003;15(10):984-990. doi:10.1046/J.1365-2826.2003.01088.X
